# Primary hepatic methotrexate-associated lymphoproliferative disorder associated with Epstein-Barr virus reactivation and accompanied by spontaneous necrosis: A case report

**DOI:** 10.1097/MD.0000000000031993

**Published:** 2022-11-25

**Authors:** Takahiko Omameuda, Hideyo Miyato, Naohiro Sata, Alan Kawarai Lefor

**Affiliations:** a Department of Surgery, Division of Gastroenterological, General and Transplant Surgery, Jichi Medical University, Shimotsuke, Tochigi, Japan.

**Keywords:** Epstein-Barr virus, methotrexate -associated lymphoproliferative disorder, primary hepatic lymphoma, spontaneous regression

## Abstract

Methotrexate-associated lymphoproliferative disorder (MTX-LPD) is a major complication of methotrexate (MTX) therapy that can develop in patients with rheumatoid arthritis (RA), although primary hepatic MTX-LPD is extremely rare. Discontinuation of MTX results in remission in half of the patients with MTX-LPDs and is one treatment approach.

**Patient concern:**

A 64-year-old Japanese woman suffering from rheumatoid arthritis treated with MTX presented with upper abdominal pain.

**Diagnosis:**

Pathological evaluation showed that the tumor contained geographic necrosis and proliferation of large atypical lymphocytes strongly positive for cluster of differentiation 20 (CD20) antigen with immunohistochemical staining and Epstein-Barr Virus-encoded RNA transcript by in situ hybridization. The tumor was finally diagnosed as a primary hepatic MTX-associated Epstein-Barr Virus positive B-cell LPD.

**Interventions:**

Left hepatic lobectomy was performed for diagnosis and therapy.

**Outcomes:**

No sighs of recurrence were observed for 2 years.

**Lessons:**

This patient demonstrated that MTX-LPD could arise in the liver, although it is rare. If liver tumors arise in patients taking MTX, examination of sIL-2R, Epstein-Barr virus-VCA IgG and EBNA might support the diagnosis of MTX-LPD. In this case, the primary hepatic MTX-LPD became necrotic without discontinuation of MTX. It is generally believed that withdrawal of MTX restores antitumor immunity resulting in tumor necrosis. This case indicates that spontaneous regression might occur without any treatment in some patients treated for RA with MTX-LPD. The relationship between MTX-LPD and spontaneous necrosis is unclear and further data is required to characterize the types of patients that will develop spontaneous regression without intervention.

## 1. Introduction

Methotrexate (MTX), a commonly used immunosuppressive agent, is an important modality in the management of patients with rheumatoid arthritis (RA)^[1]^ to suppress joint destruction. In 1991, Ellman et al first reported an association between lymphoma and the immunosuppression induced by MTX therapy in patients with RA.^[2]^ With the increasing frequency of MTX use in patients with RA, MTX has also been noted to cause methotrexate-associated lymphoproliferative disorder (MTX-LPD), a major complication of MTX therapy.^[2]^ Lymphoproliferative disorders (LPDs) are a group of disorders that include temporary lymphoid cell proliferations with spontaneous regression, benign lesions, borderline malignancies, and malignant lymphomas. The liver is rarely involved in MTX-LPD^[3]^and the etiology of hepatic MTX-LPDs remains largely unknown. We report primary hepatic Epstein-Barr virus (EBV)-positive MTX-LPD in a patient with RA, in whom a large hepatic tumor developed with geographic necrosis without MTX withdrawal. Spontaneous regression may occur without intervention in some patients with MTX-LPD.

## 2. Case presentation

A 64-year-old Japanese woman with a 14-year history of RA presented with upper abdominal pain. Administration of prednisolone and bucillamine was initiated for the management of RA at the time of disease onset. However, symptoms due to RA were not well controlled and MTX was started at 8mg/week, and the dose of MTX gradually increased to 12.5mg/week. Infliximab was started at 250mg/4 weeks at the same time. For the past 10 years, the symptoms have been controlled with MTX at 12.5mg/week, infliximab at 250mg/4weeks and prednisolone at 5mg/day. She had no family history of liver diseases or collagen diseases.

**Figure 1.**
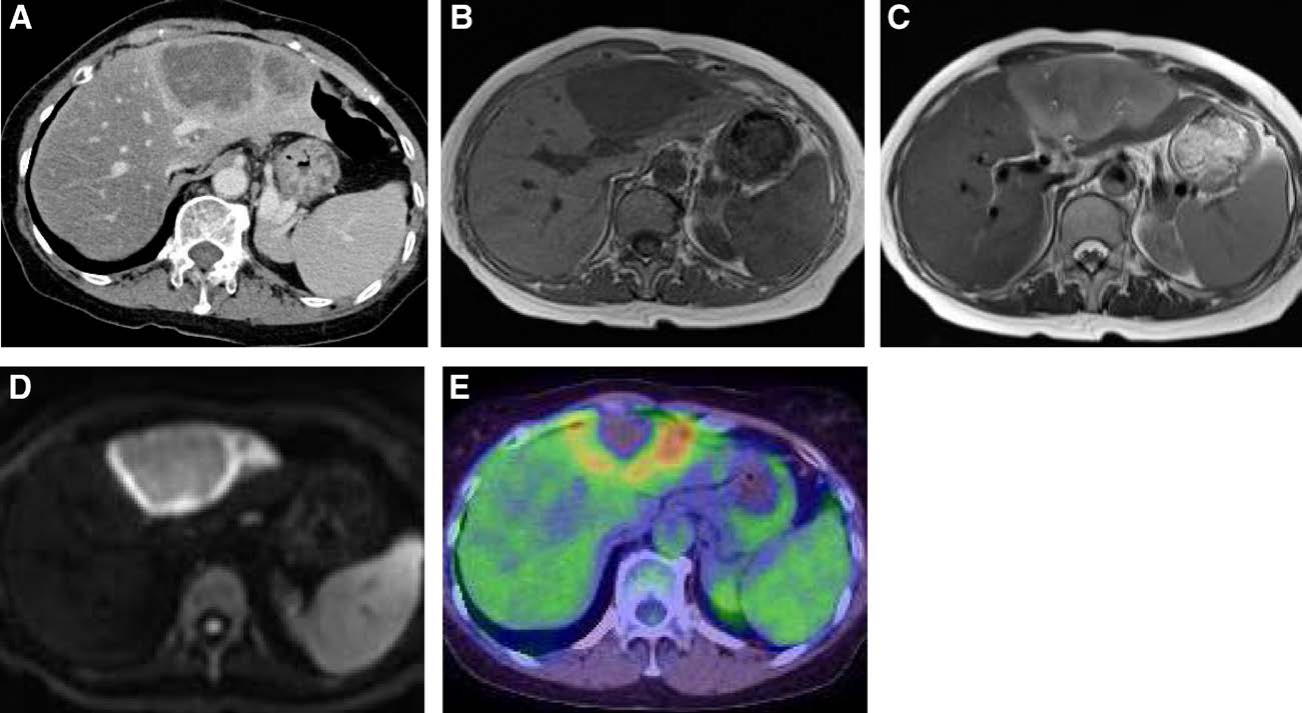
Abdominal contrast-enhanced computed tomography (CT) scan (A), magnetic resonance imaging (MRI) (B, C, D) and 18F-fluorodeoxyglucose positron emission tomography CT (FDG-PEG/CT) (E) in the present patient. Enhanced CT scan in the late phase shows annular enhancement at the tumor edge and a low-density area in the center of the tumor (A). MRI showed low intensity of the tumor on T1-weighted images (B), faint high intensity on T2-weighted images (C), and high intensity at the tumor edge on diffusion images (D). FDG-PEG/CT scan images showed abnormal accumulation of FDG mainly at the tumor edge in the lateral segment [maximum standardized uptake value, standardized uptake value max (SUVmax) = 8.22] (E). CT = computed tomography, FDG-PEG = fluorodeoxyglucose positron emission tomography, MRI = magnetic resonance imaging.

Contrast-enhanced computed tomography (CT) scan of the abdomen was performed to evaluate the abdominal pain, which revealed a single 90mm nodular mass in the left lobe of the liver. A dynamic study revealed mild annular enhancement and a low-density area in the center of the tumor (Fig. 1A). Magnetic resonance imaging (MRI), showed that the tumor was low intensity on T1-weighted, faint high intensity on T2-weighted and high intensity on diffusion images (Fig.1B–D). 18F-fluorodeoxyglucose positron emission tomography/CT (FDG-PEG/CT) scan showed abnormal accumulation mainly at the tumor margin in the left lateral segment (the maximum standardized uptake value [SUVmax] = 8.22), and no abnormal accumulation at other sites such as the spleen, lymph nodes, or bone marrow (Fig.1E). Laboratory studies revealed elevated levels of soluble interleukin-2 receptor (sIL-2R, 2340U/mL). In contrast, levels of serum tumor markers, such as alpha-fetoprotein and protein induced by vitamin K absence or antagonist-II (PIVKA-II), were within normal limits. Serum levels of liver enzymes were slightly elevated. Tests for viral markers revealed that hepatitis B surface antigen was negative, hepatitis C antibody negative, and anti-EBV viral capsid antigen antibody immunoglobulin G (IgG) antibody (EBV-VCA IgG) and EBV anti-Epstein-Barr nuclear antigen (EBNA) titers were elevated (Table 1). The preoperative differential diagnoses included intrahepatic cholangiocarcinoma, hepatocellular carcinoma and hepatic malignant lymphoma; however, no definitive diagnosis could be made. We initially considered performing a biopsy but were concerned about the possibility of this leading to tumor dissemination and performed left lobectomy for both diagnosis and treatment.

**Table 1:** Preoperative blood test results.

Peripheral blood		Biochemistry		Rheumatoid factor	7.3U/mL
White blood cells	3.2 × 10³/μL	C-reactive protein	0.13mg/dL	Anti-CCP antibody	3.9U/mL
Neutrophils	78.10%	Total protein	6g/dL	MMP-3	137.9ng/mL
Eosinophils	0.10%	Albumin	3.9g/dL	sIL-2R	2340U/mL
Basophils	0.50%	Total bilirubin	1.06mg/dL	HBs-Ag	(-)
Monocytes	5.20%	Alkaline phosphatase	253IU/L	HCV-Ab	(-)
Lympocytes	16.10%	γ-gulutamyl transpeptidase	28IU/L	HIV-Ab	(-)
Red blood cells	431 × 10^4^/μL	Aspartate aminotransferase	42IU/L	Tumor markers	
MCV	90.4fL	Alanine aminotransferase	35IU/L	PIVKA-Ⅱ	18AU/mL
MCH	29.9pg	Lactate dehydrogenase	216IU/L	CEA	1.4ng/mL
MCHC	33.00%	Blood-urea-nitrogen	12mg/dL	CA19-9	31U/mL
Hemoglobin	12.9g/dL	Creatinine	0.53mg/dL	AFP	8ng/mL
Hematocrit	38.90%	Ureic acid	3.8mg/dL	Virus titer	
Platelet count	13.3 × 10^4^/μL	Na	144mEq/L	EBV-VCA IgG	16fold
Coagulation		K	3.7mEq/L	EBV-VCA IgM	<10fold
PT-INR	1.12	Cl	108mEq/L	EBV-EBNA	1fold
APTT	33.5seconds	Ferritin	212.3ng/mL		

AFP = alpha-fetoprotein, anti-CCP = antibody anti-cyclic citrullinated peptide antibody, APTT = activated partial thromboplastin time, CA19-9 = carbohydrate antigen 19-9,CEA = carcinoembryonic antigen, EBV-EBNA = Epstein–Barr virus-Epstein–Barr virus nuclear antigen, EBV-VCA = Epstein–Barr virus-viral capsid antigen, HBs Ag = hepatitis B surface antigen, HCV-Ab = hepatitis C virus antibody, HIV-Ab = human immunodeficiency virus antibody, IgA = immunoglobulin A, IgG = immunoglobulin G, IgM = immunoglobulin M, MCV = meen corpuscular volume, MCH = mean corpuscular hemoglobin, MCHC = mean corpuscular hemoglobin concentration, MMP-3 = matrix metalloproteinase 3, PIVKA-II = protein induced by vitamin K absence-II, PT-INR = prothrombintime-international normalized ratio, sIL-2R = soluble interleukin-2 receptor.

**Figure 2.**
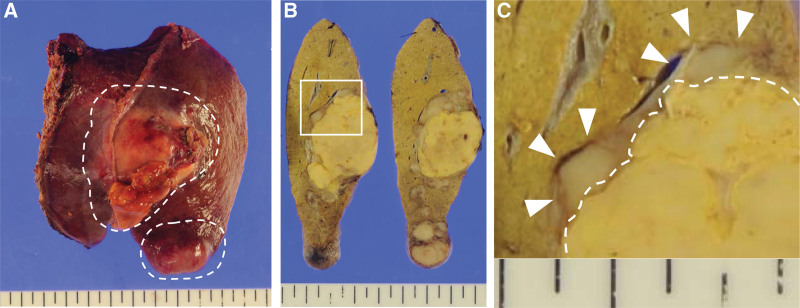
(A) The white dotted lines indicate 2 yellow-white, well-defined masses. (B) These 2 tumors were contiguous within the liver. (C) Enlarged view of white frame of Figure 2B. White arrows indicate white solid components of the tumor edge and white dotted lines indicate the tumor central area composed of yellow soft tissue.

**Figure 3.**
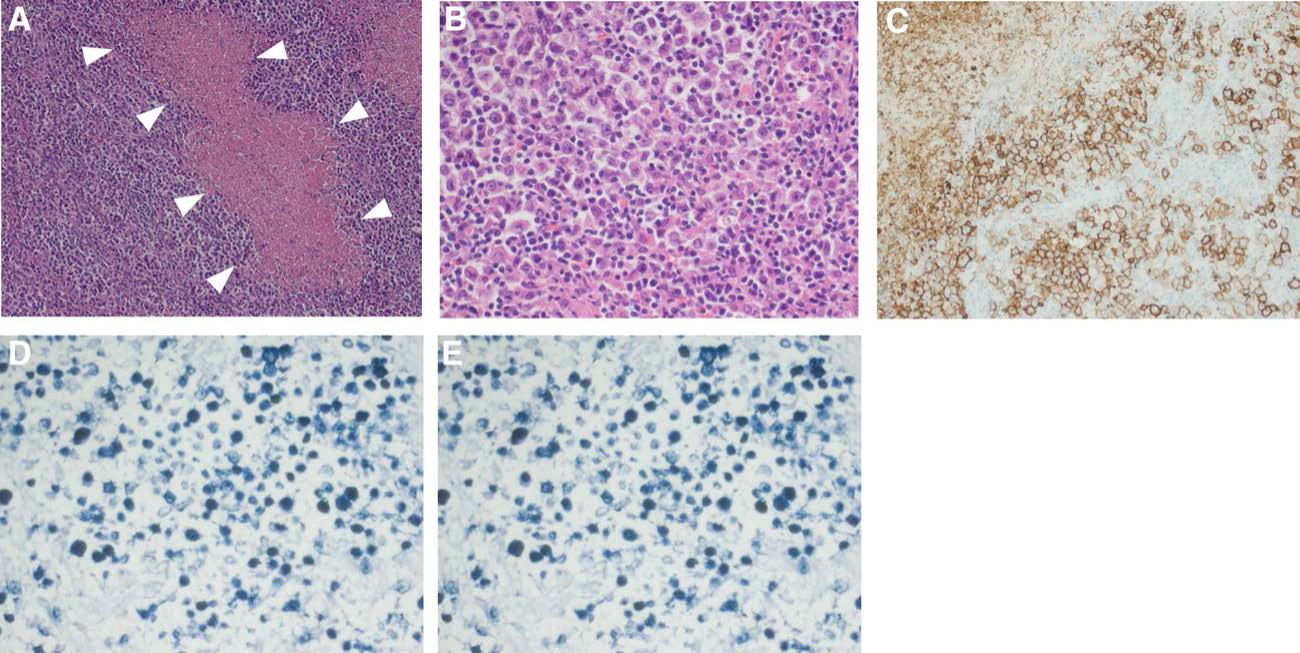
Histopathology of the liver tumor. (A) White arrows indicate geographic necrosis in the tumor (H&E stain). (B) Large atypical lymphocytes proliferated in the tumor (H&E stain). (C) immunohistochemical staining with anti-CD20 antibody. (D) immunohistochemical staining with anti-CD3 antibody. (E) the detection of EBV-encoded small RNAs (EBER) by in situ hybridization (ISH). Tumor cells were positive for CD20, EBER and negative for CD3. The tumor was diagnosed as an EBV-positive B-cell lymphoma.

The tumor was grossly composed of 2 well-defined mass lesions, which were contiguous within the liver (Fig.2A–B). White solid components at the tumor edge encircled the broad central area composed of yellow soft tissue (Fig.2B–C). Histopathologic findings showed large atypical lymphocytes proliferating at the tumor edge, with geographic necrosis spreading in the central area (Fig.3A–B). Lymphocytes were strongly positive for cluster of differentiation 20 (CD20) antigen, negative for CD3 by immunohistochemical staining and positive for EBV-encoded RNA transcript (EBER) by in situ hybridization (Fig.3C–E). Based on the history of oral administration of MTX and reactivation of EBV infection, the tumor was finally diagnosed as a primary hepatic EBV-positive B-cell MTX-LPD. According to the World Health Organization classification, this tumor belongs to “other iatrogenic immunodeficiency-associated lymphoproliferative disorders.” Serum levels of sIL-2R and serum level of liver enzymes decreased to within the normal range after MTX withdrawal. No signs of recurrence were observed for 2 years.

## 3. Discussion

MTX is widely used to treat patients with RA. LPDs develop in patients with autoimmune diseases receiving immunosuppressive agents such as MTX, which is associated with other iatrogenic immunodeficiency-associated LPDs. According to the World Health Organization classification of tumors of hematopoietic and lymphoid tissues, these disorders are categorized as lymphoid proliferations or lymphomas that arise in patients treated with immunosuppressive drugs for autoimmune diseases or conditions other than in post-transplant settings.^[3]^

Primary hepatic lymphoma (PHL) is very rare, accounting for just 0.4% of extra-nodal non-Hodgkin’s lymphomas.^[3,4]^ Although MTX-LPDs can arise in various extra-nodal organs, primary hepatic lesions are extremely rare. To date, only 12 patients with liver MTX-LPD have been reported. Nine patients had multiple lesions compared to three with single lesions.^[5]^ Five patients had lesions outside the liver, including the lung (n = 2), spleen (n = 2), abdominal para-aortic lymph nodes (n = 2), adrenal glands (n = 2), cervical lymph nodes (n = 1), and retroperitoneal lymph node (n = 1). Seven patients had lesions restricted to the liver. In the present patient, there was a single hepatic lesion. According to a report by Ono et al,^[5]^ liver function tests were slightly elevated, whereas sIL-2R, LDH, and CRP were more elevated in patients with liver MTX-LPD. In the present patient, liver function tests were slightly elevated, and sIL-2R levels were elevated, and LDH and CRP levels were not elevated. Imaging examinations of patients with PHL display various findings, including solitary, multiple, or infiltrative patterns, and there are no definitive diagnostic features.^[2]^ It is difficult to distinguish PHL from hepatocellular carcinoma or metastatic tumors and to obtain a correct diagnosis based on imaging studies alone.^[6]^ Characteristic imaging findings with hepatic MTX-LPD have not been delineated because of their rarity. Therefore, biopsy or surgical resection of suspected PHL and subsequent histological examination is necessary to make a definitive diagnosis.^[7]^ In the present patient, there was a single hepatic lesion and a definitive diagnosis could not be achieved based on imaging studies. Surgical resection was performed for diagnostic and therapeutic purposes. The development of MTX-LPD in various organs in patients taking MTX must be considered.

Immunocompromised patients have a higher risk of developing LPD than healthy individuals.^[8]^ A number of recent reports have shown that patients suffering from RA treated with immunosuppressive agents, including MTX, have high prevalence of LPDs.^[9-11]^ RA itself is associated with increased risk of lymphoma by 2.5 times compared to healthy people.^[12]^ The extent to which MTX contributes to a further increased risk of developing LPDs remains unclear. It is well known that immunosuppression predisposes patients to the development of EBV-associated lymphomas. EBV infection was detected in 12- 44% of lymphoma tissues that have emerged in patients with RA.^[7]^ It was reported that reactivation of EBV was detected in most patients with MTX-LPDs, and LPDs regressed in half of them after discontinuation of MTX therapy.^[11]^ Immunosuppression induced by MTX may be involved in the reactivation of EBV, which may lead to the development of MTX-LPD.^[13]^ In the present patient, serological tests for EBV-VCA IgG and EBNA were positive, and the test for EBV IgM was negative. These results indicate reactivation of EBV. Pathological specimens showed that the atypical large lymphoid cells infiltrating the tumor were positive for CD20 and EBVR. These results taken together led to the diagnosis of primary hepatic EBV-positive B-cell MTX-LPD.

There is no established standard treatment for patients with MTX-LPD.^[14]^ Salloum et al reported that 62.5% of patients showed at least partial regression of the LPD in response to MTX withdrawal without additional antitumor therapy, and most of these patients were EBV-positive.^[15]^ If hepatic MTX-LPD is suspected based on the findings of oral MTX administration, EBV reactivation (elevation of EBV-VCA IgG and EBNA titers) and elevation of sIL-2R, pathological examination using a liver biopsy should be considered to avoid surgical intervention. If a definitive diagnosis is achieved, withdrawal of MTX with observation is 1 treatment option. In some cases, regression of liver tumors may not occur after MTX withdrawal, or the tumor might relapse even if withdrawal of MTX induces temporary regression of MTX-LPD. Chemotherapy administration should be considered in such patients.

It is generally believed that the withdrawal of MTX restores antitumor immunity with resulting tumor necrosis. In other situations, necrosis can be attributed to the effects of chemotherapy or other treatment. In the present patient, pathological examination of the resected specimen revealed geographic necrosis in the tumor without MTX withdrawal. Ohkura et al reported that primary adrenal MTX-LPD became necrotic without any treatment, showing the possibility of tumor resolution without intervention.^[16]^ Similar to the present patient, the patient in that report was EBER-positive and EBNA-positive.

In EBV-infected lymphoma or lymphoproliferation, anti-EBV immune responses are believed to induce responses against lymphoma resulting in spontaneous regression.^[17]^ EBV-transformed B lymphocytes and EBV-infected lymphoma cells produce IL-12, a cytokine that promotes cellular immunity.^[18]^ IL-12 production by lymphoma cells may be involved in the spontaneous regression of EBV-infected lymphoma or lymphoproliferative disorders. A recent study that compared differences in gene analysis profiles and immunohistochemistry between DLBCL developing during MTX administration and DLBCL in the general population revealed a microenvironment with high numbers of cytotoxic T lymphocytes and M2 macrophages in MTX-DLBCL.^[19]^ Antitumor immune responses might occur concurrently with immunosuppression induced by MTX in some cases of MTX-LPD. Spontaneous regression has been observed in several types of malignant tumors, including low-grade non-Hodgkin’s lymphoma and chronic lymphocytic leukemia.^[20]^ These tumors appear to display spontaneous regression more frequently than other malignancies. MTX-LPD might be susceptible to spontaneous regression without intervention.

## 4. Conclusion

A patient with primary hepatic EBV-positive MTX-LPD which underwent spontaneous necrosis without MTX withdrawal is reported. The relationship between MTX-LPD and spontaneous necrosis is unclear and further studies are required to characterize the types of tumors that will spontaneously regress without intervention. Understanding of the mechanisms associated with spontaneous regression may lead to development of new therapeutic approaches.

## Acknowledgments

The authors thank patients for their participation in this study.

## Author contributions

All authors have read and agree to publication of the manuscript.

**Conceptualization:** Takahiko Omameuda, Hideyo Miyato.

**Drafting the manuscript:** Takahiko Omameuda, Hideyo Miyato.

**Resources:** Takahiko Omameuda, Hideyo Miyato.

**Supervision:** Hideyo Miyato.

**Writing – original draft:** Takahiko Omameuda, Hideyo Miyato.

**Writing – review & editing:** Hideyo Miyato, Naohiro Sata, Alan Kawarai Lefor.
